# Role of flow of information in the speedup of quantum evolution

**DOI:** 10.1038/s41598-018-34890-x

**Published:** 2018-11-15

**Authors:** J. Wang, Y. N. Wu, Z. Y. Xie

**Affiliations:** School of physics and technology, University of Jinan, Jinan, 250022 China

## Abstract

Quantum evolution can be accelerated in a non-Markovian environment. Previous results show that the formation of a system-environment bound state governs the quantum speedup. Although a stronger bound state in the system-environment spectrum may seem like it should cause greater speed of evolution, this seemingly intuitive thinking may not always be correct. We illustrate this by investigating a classical-driven qubit interacting with a photonic crystal waveguide in the presence of a mirror, resulting in non-Markovian dynamics for the system. Within the considered model, we show the influence of the mirror and the classical field on the evolution speed of the system. In particular, we find that the formation of a bound state is not the essential reason for the acceleration of evolution. The quantum speedup is attributed to the flow of information, regardless of the direction in which the information flows. Our conclusion can also be used in other non-Markovian environments.

## Introduction

Quantum evolution speed determines how quickly a quantum system needs to evolve between an initial state and a target state in a given process. Realization of controllable speeding up of evolution of a quantum system plays a key role in many technological applications, such as suppressing decoherence^1^ and improving the efficiency of quantum computation^2,3^. For closed quantum systems, it has been shown that the entanglement can accelerate the quantum evolution^4,5^. Owing to the inevitable interaction between any system and its environment, a considerable amount of work has witnessed research on controlling speedup in more general open systems recently. One important discovery is that the non-Markovian process induced by the memory effect of environment can induce dynamical acceleration^6,7^, and therefore lead to a smaller quantum speed limit time (QSLT), which is defined as the minimal evolution time between two states^8,9^. This phenomenon has been proven by the experiment in cavity quantum electrodynamics (QED) systems^10^.

Much effort has been made to explore how to exploit the non-Markovian environment itself to speed up quantum evolution. Some methods have been provided to speed up quantum evolution for open systems, such as by engineering multiple environments^11^, driving the system by an external classical field^12^, and using the periodic dynamical decoupling pulse^13^. The reason for quantum speedup using the above methods is found to be the increase of the degree of non-Markovianity. Recently, the authors of ref.^14^ showed that both the non-Markovianity and the quantum speedup are attributed to the formation of a system-environment bound state, i.e., the stationary state of the entire system with eigenvalues residing in the band gap of energy spectrum^15–17^. If the bound state is established, the evolution of the system becomes non-Markovian, and thus quantum speedup occurs. A good example of this is the situation in which a two-level atom is coupled to an environment with a Ohmic spectrum. For this model, it has been found that providing stronger bound states can lead to a higher degree of non-Markovianity, and hence to greater speed of quantum evolution^14^. Based on this monotonic relation between the three, controlling speedup through manipulation of a system-environment bound state has recently been studied^18^. In some sense, one may intuitively think that the formation of a bound state can be seen as the essential reflection to the speedup of quantum evolution. However, the mechanism for quantum speedup in non-Markovian quantum systems is still poorly understood if the environment is too complex.

The purpose of this paper is to examine the relationship between the formation of a bound state, non-Markovianity, and quantum speedup. To do so, in this paper, we focus on the problem of quantum speedup in a photonic crystal (PC) waveguide^19,20^ with one mirror, for a classical-driven qubit. The dynamics of this quantum system are non-Markovian^21^, due to the emitted light bouncing back and forth between the mirror and the qubit. This structure has been used to develop single-photon transistors^22^ and atomic light switches^23^. In this setting, the speedup process for the embedded qubit can be acquired by manipulation of the classical driving field and the memory time of the environment. Regarding the mechanism of quantum speedup, some unexpected and nontrivial results are found. The formation of a bound state can indeed lead to non-Markovian evolution, but it does not necessarily result in quantum speedup. The speedup of quantum evolution is attributed to the flow of information, regardless of the direction of information flows. We illustrate that it is not the amount of backflow information, i.e., the non-Markovianity, but rather the information flow volume that ultimately determines the actual speed of quantum evolution.

In the following, we first present our physical model and construct the measure of the actual speed of quantum evolution based on an information geometric formalism. We then use this measure to investigate how the environment affects the speed of quantum evolution within our model. To clarify the mechanism for quantum speedup, we first explore the interrelationship between the formation of a bound state, non-Markovianity, and quantum speedup, and then present the role of the flow of information in the speedup of evolution.

## Results

### Physical model

**Figure 1:**
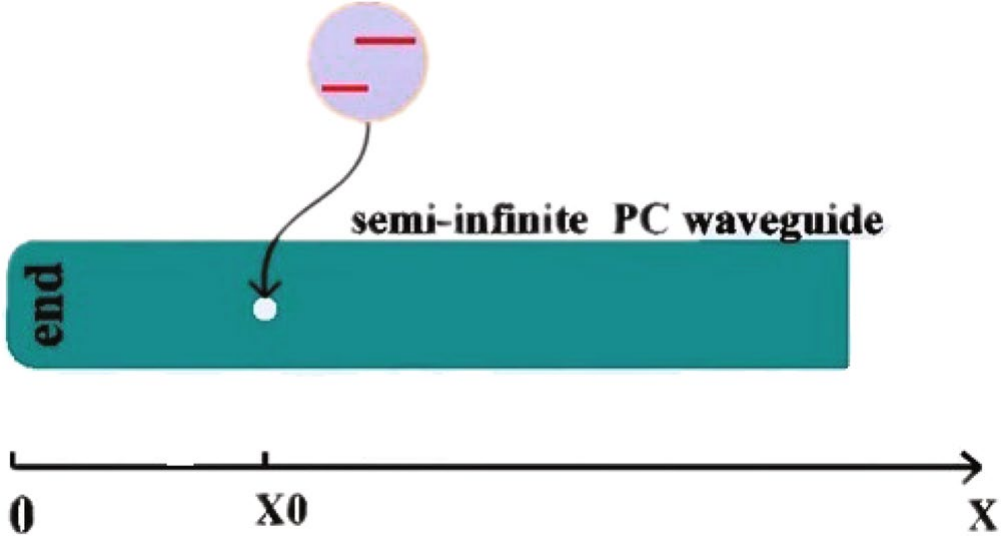
The implementation of the model. A single-end PC waveguide, whose end lies at *x* = 0, is coupled to a two-level atom (qubit) at *x* = *x*_0_.

We consider a qubit (two-level atom with excited and ground states |*e*〉 and |*g*〉) with frequency *ω*_0_ driven by a classical field with frequency *ω*_*L*_. The qubit is embedded in a PC waveguide along the *x* axis (see Fig. 1). The distance between the qubit and the waveguide end is *x*_0_. The total Hamiltonian is given by (*ħ* = 1)

1 $$H=\frac{{\omega }_{0}}{2}{\sigma }_{z}+\sum _{k}{\omega }_{k}{a}_{k}^{\dagger }{a}_{k}+{\rm{\Omega }}({e}^{-i{\omega }_{L}t}{\sigma }_{+}+H.\,c.)+\sum _{k}({g}_{k}{a}_{k}^{+}{\sigma }_{-}+H.\,c\mathrm{.}),$$

in which Ω is the driving strength of the classical field and *σ*_±_ denotes the inversion and raising operators of the qubit; ${a}_{k}^{\dagger }$ and *a*_*k*_ are the creation and annihilation operators of a photon in the *k*th mode, while *g*_*k*_ stands for the corresponding coupling.

In practice, the end of the waveguide can be considered a perfect mirror, which can lead to form the system-environment bound state^24^ and giant Lamb shift^25^. In this case, the photon dispersion relationship around the atomic frequency is of the form^26^

2 $${\omega }_{k}={\omega }_{0}+\upsilon (k-{k}_{0}),$$

where *υ* denotes the group velocity of the photon and *k*_0_ is the wave vector corresponding to *ω*_0_, i.e., *ω*_*k*0_ = *ω*_0_. The coupling strength *g*_*k*_ can be given by^27^

3 $${g}_{k}=\sqrt{{\rm{\Gamma }}\upsilon /\pi }\,\sin \,k{x}_{0}$$

with the atomic spontaneous emission rate Γ. For convenience of calculations, we first give the effect Hamiltonian of our model. By using the unitary transformation *U* = exp(−*iω*_*L*_*σ*_*z*_*t*/2), the Hamiltonian in Eq. (1) can be transformed into

4 $$H=\frac{{\rm{\Delta }}}{2}{\sigma }_{z}+{\rm{\Omega }}{\sigma }_{x}+\sum _{k}{\omega }_{k}{a}_{k}^{\dagger }{a}_{k}+\sum _{k}({g}_{k}{a}_{k}^{+}{\sigma }_{-}{e}^{-i{\omega }_{L}t}+H.\,c\mathrm{.)},$$

where Δ = *ω*_0_ − *ω*_*L*_. In the basis $\{|\,+\,\rangle =\cos \,\frac{\eta }{2}|e\rangle +\sin \,\frac{\eta }{2}|g\rangle ,|\,-\,\rangle =-\,\sin \,\frac{\eta }{2}|e\rangle +\cos \,\frac{\eta }{2}|g\rangle \}$ with $\eta ={\tan }^{-1}\mathrm{(2}|{\rm{\Omega }}|/{\rm{\Delta }})$, the first two terms on the right-hand side of the above equation can be diagonalized, and then the effect Hamiltonian can be rewritten as

5 $${H}_{eff}=\frac{{\omega }_{ef}}{2}{\xi }_{z}+\sum _{k}{\omega }_{k}{a}_{k}^{\dagger }{a}_{k}+\sum _{k}({G}_{k}{a}_{k}^{+}{\xi }_{-}+H.\,c\mathrm{.)},$$

where ${\omega }_{ef}=\sqrt{{{\rm{\Delta }}}^{2}+4{|{\rm{\Omega }}|}^{2}}$, ${G}_{k}={\cos }^{2}\frac{\eta }{2}{g}_{k}$, and ${\xi }_{+}={\xi }_{-}^{\dagger }=|\,+\,\rangle \langle \,-\,|$ and ${\xi }_{z}=|\,+\,\rangle \langle \,+\,|-|\,-\,\rangle \langle \,-\,|$.

Supposing that the initial state of the joint atom-field system is of the form $|\phi (0)\rangle =|\,+\,\rangle |\tilde{0}\rangle$, with $|\tilde{0}\rangle$ denoting the state of vacuum reservoirs, after time *t* > 0 this initial state evolves into the state

6 $$|\phi (t)\rangle ={c}_{+}(t)|+,\tilde{0}\rangle +\sum _{k}{c}_{k}(t)|-,{\tilde{1}}_{k}\rangle ,$$

in which $|{\tilde{1}}_{k}\rangle$ is the state of the reservoir with exactly one photon in mode *k*. With the help of the Schrödinger equation, we obtain the equations of motion for the amplitudes, which are governed by:

7 $${\dot{c}}_{+}(t)=-\,i\sum _{k}{G}_{k}{e}^{i({\omega }_{ef}-{\omega }_{k})t}{c}_{k}(t),$$

8 $${\dot{c}}_{k}(t)=-\,i{G}_{k}{c}_{+}(t){e}^{-i({\omega }_{ef}-{\omega }_{k})t}.$$

By formally integrating the last equation (8) and substituting this into Eq. (7), we obtain:

9 $${\dot{c}}_{+}(t)=-\,{\int }_{0}^{t}dt^{\prime} g(t-t^{\prime} ){c}_{+}(t^{\prime} )$$

with $g(t-t^{\prime} )=\sum _{k}{|{G}_{k}|}^{2}{e}^{i({\omega }_{ef}+{\omega }_{L}-{\omega }_{k})(t-t^{\prime} )}$. Through integration of the correlation function $g(t-t^{\prime} )$ over *k* and replacing it in Eq. (9), we obtain:

10 $${\dot{c}}_{+}(t)=-\,{\cos }^{4}\frac{\eta }{2}\frac{{\rm{\Gamma }}}{2}{c}_{+}(t)+{\cos }^{4}\frac{\eta }{2}\frac{{\rm{\Gamma }}}{2}{e}^{i({\omega }_{ef}-{\rm{\Delta }}){t}_{d}}{e}^{i\varphi }{c}_{+}(t-{t}_{d})\,{\rm{\Theta }}\,(t-{t}_{d}),$$

where we have defined the phase *ϕ* = 2*k*_0_*x*_0_ and used the Heaviside step function Θ(*t*). *t*_*d*_ = 2*x*_0_/*υ* denotes the time the photon needs for the distance atom-mirror-atom. This time can be interpreted as the memory time of the reservoir^27^. Performing the Laplace transformation of Eq. (10), we obtain:

11 $${\tilde{c}}_{+}(s)=\frac{1}{s+{cos}^{4}\frac{\eta }{2}\frac{{\rm{\Gamma }}}{2}-{\cos }^{4}\frac{\eta }{2}\frac{{\rm{\Gamma }}}{2}{e}^{i({\omega }_{x}{t}_{d}+\varphi )}{e}^{-s{t}_{d}}},$$

where *ω*_*x*_ = *ω*_*ef*_ − Δ. By inverting the Laplace transform, we can obtain^28^

12 $$\begin{array}{c}{c}_{+}(t)={e}^{-{\cos }^{4}\frac{\eta }{2}\frac{{\rm{\Gamma }}}{2}t}\sum \frac{1}{n!}{({\cos }^{4}\frac{\eta }{2}\frac{{\rm{\Gamma }}}{2}{e}^{i{\omega }_{x}{t}_{d}}{e}^{i\varphi }{e}^{{\cos }^{4}\frac{\eta }{2}\frac{{\rm{\Gamma }}}{2}{t}_{d}})}^{n}\cdot {(t-n{t}_{d})}^{n}\,{\rm{\Theta }}\,(t-n{t}_{d}),\end{array}$$

where the dynamical evolution is witnessed by the memory time *t*_*d*_.

### Measure of dynamical speed

The dynamical speed and the QSLT can be quantified by different measures, such as the Taddei *et al*.^29^ measure based on the quantum Fisher information metric, the del Campo *et al*.^30^ measure based on the relative purity, and the Paiva-Pires *et al*.^31^ measure by adopting the information geometric approach using the Wigner-Yanase (WY) information metric. The measure based on the WY information metric can lead to a tighter QSLT, expecially for amplitude damping dynamics.

Following ref.^31^, we briefly give the formal derivation of the actual dynamical speed corresponding to the model described in the preceding section. Based on the information geometric formalism^32^, the set of quantum states is indeed a Riemannian manifold; that is, the set of density operators over the Hilbert space. The geometric length between the given initial state *ρ*_0_ and the final state *ρ*_*τ*_ can be naturally measured by using possible Reimannian metrics over the manifold. According to the theorem of the Morozova-Čencov-Petz theorem^33–35^, any monotonic Riemannian metric can be given by the unified form:

13 $${g}^{f}(A,B)=\frac{1}{4}Tr(Ac({L}_{\rho },{R}_{\rho })B),$$

where *A* and *B* are any Hermitian operators, and *c*(*x*, *y*) is a symmetric function defined as:

14 $$c(x,y)=\frac{1}{yf(x/y)}$$

with *f*(*t*) being the Morozova-Čencov (MC) function that fulfills *f*(*t*) = *tf*(1/*t*) and *f*(1) = 1^36^. The MC function is related to our chosen Riemannian metric, in which different forms of MC functions denote different Reimannian metrics. *L*_*ρ*_ and *R*_*ρ*_ are two linear super-operators defined as *L*_*ρ*_*A* = *ρA* and *R*_*ρ*_ *A* = *Aρ*.

Given the unified form of a Riemannian metric, the squared infinitesimal length between two neighboring quantum states *ρ* and *ρ* + *dρ* can be given by^37^

15 $$d{s}^{2}={g}^{f}(d\rho ,d\rho ).$$

Here, we consider a dynamical evolution with a map Λ_*t*_. The evolved state is *ρ*_*t*_ = Λ_*t*_*ρ*_0_ with a initial state *ρ*_0_. Along the evolved path between *ρ*_0_ and *ρ*_*τ*_ with *t* ∈ [0, *τ*], the line element of the path can be expressed as:

16 $$dl=\sqrt{{g}^{f}({\partial }_{t}{\rho }_{t},{\partial }_{t}{\rho }_{t})}dt.$$

Then, the instantaneous speed of quantum evolution can be given by:

17 $$V=\frac{dl}{dt}=\sqrt{{g}^{f}({\partial }_{t}{\rho }_{t},{\partial }_{t}{\rho }_{t})}.$$

The average speed between time zero and *τ* is:

18 $${V}_{a}=\frac{1}{\tau }{\int }_{0}^{\tau }Vdt.$$

The above equation can be used to evaluate the actual speed of quantum evolution.

In order to obtain the measure of dynamical speed in an explicit form, we can rewrite the evolved state *ρ*_*t*_ in the form of its spectral decomposition, $\,{\rho }_{t}\,=\,\sum _{k}{p}_{k}|{\varphi }_{k}\rangle \langle {\varphi }_{k}|\,$, with 0 < *p*_*k*_ < 1 and $\sum _{k}{p}_{k}\,=\,1$. According to the Morozova-Čencov-Petz formalism, the instantaneous speed can be rewritten as^32^

19 $$V=\sqrt{\sum _{k}\frac{{|{\dot{p}}_{k}|}^{2}}{4{p}_{k}}+\sum _{k\ne l}c({p}_{k},{p}_{l})\frac{{p}_{k}({p}_{k}-{p}_{l})}{2}{|\langle {\varphi }_{l}|{\dot{\varphi }}_{k}\rangle |}^{2}}.$$

Clearly, any contractive Riemannian metric can be employed to evaluate the speed of evolution with a different type of MC function *f*(*t*). As shown in ref.^36^, a generic MC function must fulfill *f*_min_(*t*) < *f*(*t*) < *f*_max_(*t*), where *f*_min_(*t*) = 2*t*/(1 + *t*) and *f*_max_(*t*) = (1 + *t*)/2. Interestingly, an intermediate MC function with ${f}_{WY}(t)={\mathrm{(1}+\sqrt{t})}^{2}\mathrm{/4}$ and ${c}_{WY}(x,y)=\mathrm{4/}{(\sqrt{x}+\sqrt{y})}^{2}$ is that corresponding to the WY information metric, which is widely used in detecting the speed of dynamical evolution^38^. In what follows, we focus on the WY information metric.

### Controllable of quantum speedup

Here, we apply the measure constructed above to the waveguide system, and study the mechanism for controllable speedup. In order to investigate the dynamics of the system, we express the reduced density matrix of the qubit in the basis $\{|\,+\,\rangle ,|\,-\,\rangle \}$ as

20 $${\rho }_{a}(t)=(\begin{array}{cc}{\beta }^{2}{P}_{t} & \beta \sqrt{1-{\beta }^{2}}\sqrt{{P}_{t}}\\ \beta \sqrt{1-{\beta }^{2}}\sqrt{{P}_{t}} & 1-{\beta }^{2}{P}_{t}\end{array}),$$

where *P*_*t*_ = |*c*_+_(*t*)|^2^ denotes the excited-state population of the qubit. Here, we have supposed the initial state of the qubit is $|{\rm{\Psi }}(0)\rangle =\beta |\,+\,\rangle +\sqrt{1-{\beta }^{2}}|\,-\,\rangle$ (0 ≤ *β* ≤ 1). The spectral decomposition of *ρ*_*a*_(*t*) can be expressed as the form

21 $${\rho }_{a}(t)=\sum _{k=\pm }{p}_{k}|{\varphi }_{k}\rangle \langle {\varphi }_{k}|,$$

with *p*_±_ = (1 ± *λ*)/2 and $$\,|{\varphi }_{\pm }\rangle =({\alpha }_{\pm }|\,+\,\rangle +|\,-\,\rangle )/\sqrt{1+{\alpha }_{\pm }^{2}}$$, where $$\,\lambda =\sqrt{1-4{\beta }^{2}{P}_{t}+4{\beta }^{4}{P}_{t}^{2}}$$ and $${\alpha }_{\pm }=$$$$(2{\beta }^{2}{P}_{t}\pm \lambda \,-\,1)/(2\beta \sqrt{1-{\beta }^{2}}\sqrt{{P}_{t}})$$.

The dynamics of the qubit is fully determined by Eq. (10). Clearly, the first term on the right-hand side of Eq. (10) corresponds to the atomic spontaneous emission, while the second term represents the effect of the presence of the mirror on the atomic dynamical evolution. In the following, we analyze how the distance between the mirror and the atom, or the memory time *t*_*d*_, influences the dynamical speed of the system.

#### Small atom-mirror distance

**Figure 2:**
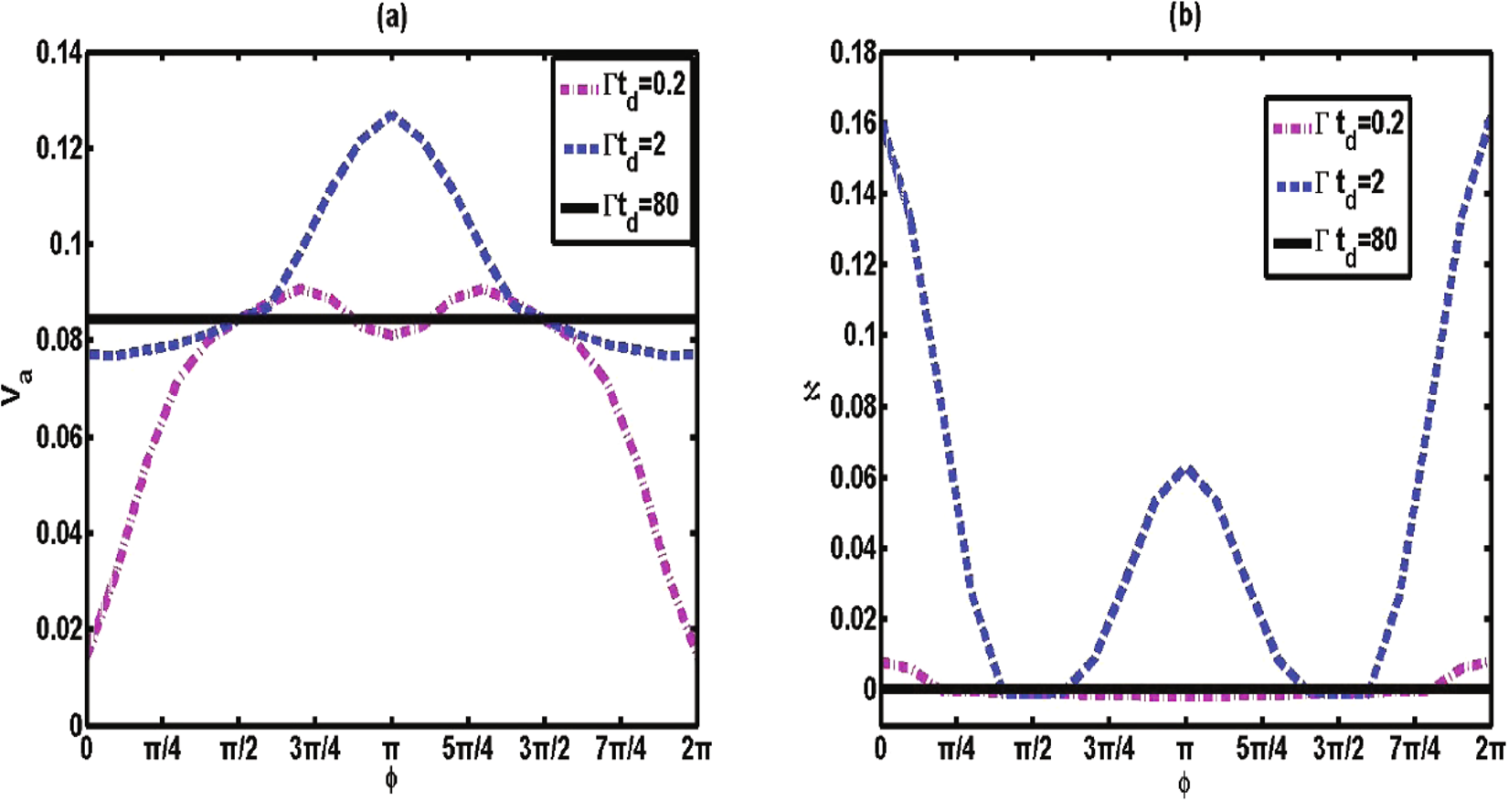
(**a**) The average speed of quantum evolution *V*_*a*_ and (**b**) the non-Markovianity ℵ between the time zero and Γ*τ* = 10 (in units of 1/Γ) as a function of phase *ϕ* for various values of memory time Γ*t*_*d*_ with Ω = 0, Δ = 0 and *β* = 1.

First, we focus on the case where the distance between the atom and the mirror *x*_0_ is small (short memory time), and ignore the influence of the classical field with Ω = 0. In this situation, Eq. (10) can be approximated as ${\dot{c}}_{+}(t)\simeq \frac{{\rm{\Gamma }}}{2}{c}_{+}(t)({e}^{i\phi }-\mathrm{1)}$. Obviously, the atomic population *P*_*t*_ will exhibit an exponential decay. Therefore, the waveguide behaves as a fully Markovian reservoir for the qubit in this case. The variation of the average speed *V*_*a*_ with respect to *ϕ* for different time *t*_*d*_ is plotted in Fig. 2(a). We can find that, in the regime where the memory time is small with Γ*t*_*d*_ = 0.2, the normalized average speed for the qubit system is always relatively small. The maximum value of *V*_*a*_ is not exceed 0.1 in the range *ϕ* ∈ [0, 2*π*].

**Figure 3:**
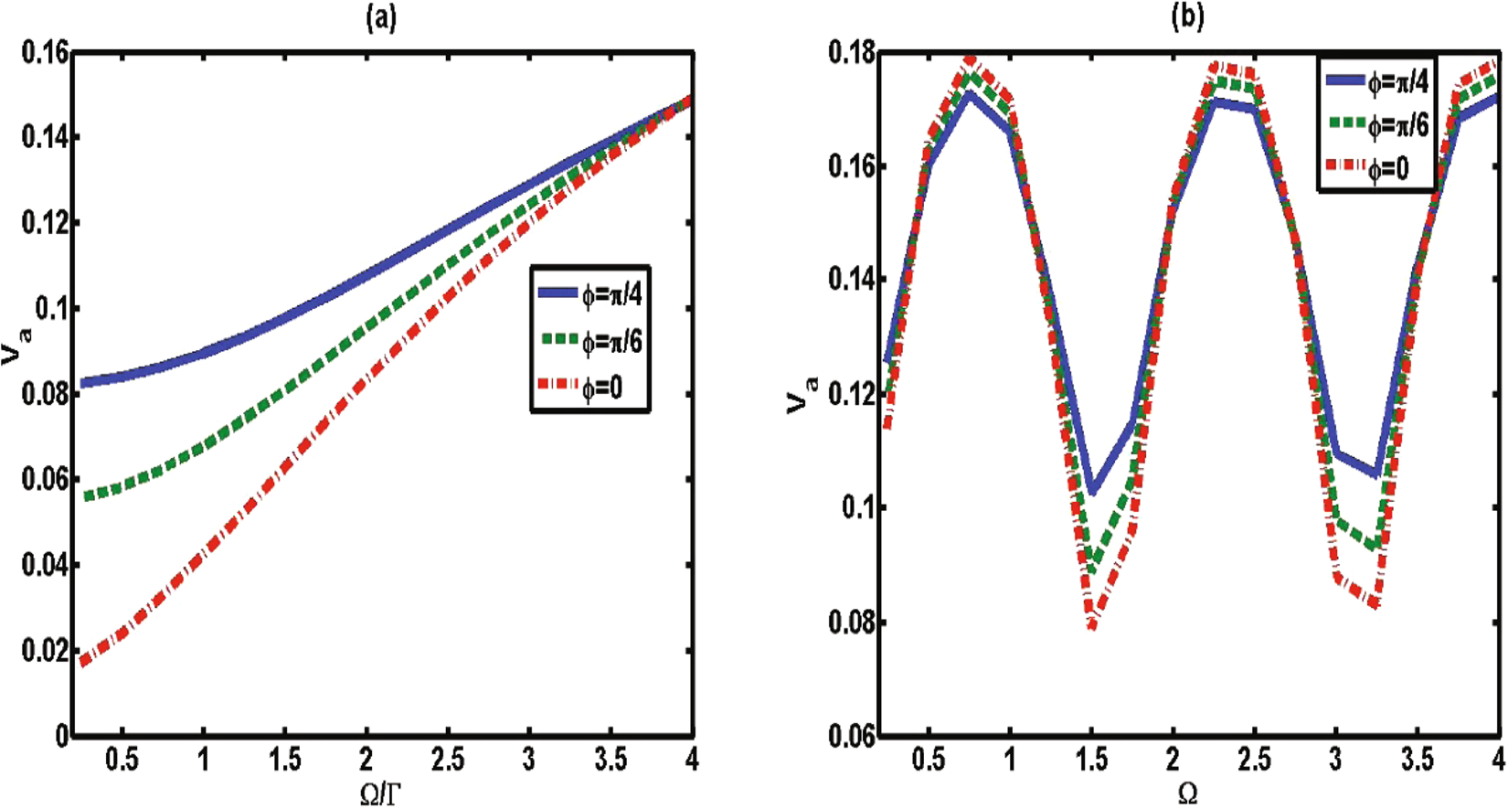
The average speed of quantum evolution *V*_*a*_ between the time zero and Γ*τ* = 10 as a function of classical driving strength Ω (in units of 1/Γ) for various values of phase *ϕ* with Δ = 0 and *β* = 1. (**a**) The memory time is small with Γ*t*_*d*_ = 0.2. (**b**) The memory time is intermediate with Γ*t*_*d*_ = 2.

To obtain the speedup of quantum evolution in this case, we show how the classical field influences the speed of the qubit. The variation of *V*_*a*_ with respect to driving strength Ω for different *ϕ* is plotted in Fig. 3(a). For each line with a fixed *ϕ*, the increase of driving strength Ω leads to an increase of the average speed. We therefore reach the interesting result that the classical field can be used to accelerate the dynamical evolution in this Markovian setting.

#### Medium length of *x*_0_

When the atom-mirror distance is in the intermediate range, the time the photon needs for a round trip (memory time *t*_*d*_) cannot be neglected. Thus, the presence of the mirror is fully responsible for the non-Markovian character. In this case, the speed of the atomic dynamical evolution is different from the above case, in which the small memory time does not induce any non-Markovianity^27^. As shown in Fig. 2(a) (dashed line), over the entire range of the phase *ϕ* the average speed in the intermediate regime (Γ*t*_*d*_ = 2) is faster than in the case in which the memory time is small. An interesting feature here is that the speed *V*_*a*_ has an obvious periodicity change under action of the driving strength [as shown in Fig. 3(b)]; that is, in the intermediate regime the speed of dynamical evolution can be controlled to a speed-up and slowed-down process by the classical driving strength.

#### Very large atom-mirror distance *x*_0_

It is worth noting the situation in which the atom is located far away from the mirror. In this case, the memory time is so large that the emitted photon cannot be reflected by the waveguide end, even when the qubit has fully decayed to the ground state. Furthermore, dynamical evolution occurs independently of the phase *ϕ* and the classical field. This is due to the fact that the interference between incoming and outgoing radiation cannot occur in the long-time limit. Thus, as expected, the average speed exhibits a plateau independent of *ϕ*, as shown in Fig. 2(a) (solid line).

Here, we present a method of accelerating the dynamical speed by manipulating the classical driving field and the memory time of the reservoir. Our proposed scheme is experimentally accessible. In the experiment, the planar PC can be prepared by a GaAs PC membrane and the qubit can be prepared by the self-assembled InGaAs quantum dots (QDs) with a lower density^39^. We can use the method of electron-beam lithography to construct the PC waveguide. Furthermore, the recent experiment has demonstrated an excellent control on the atomic embedded position by the method of electro-hydrodynamic jet printing^40,41^.

### Relationship between formation of bound state, non-Markovianity, and quantum speedup

The previous result shows that^14^ the formation of the system-environment bound state is the essential reason for the quantum speedup. It was demonstrated that providing stronger bound states can lead to a higher degree of non-Markovianity, and hence to greater speed of quantum evolution. To understand the physical reason for the speedup in our model, we further study the interrelationship between the formation of a bound state, the non-Markovianity, and the speed of quantum evolution.

**Figure 4:**
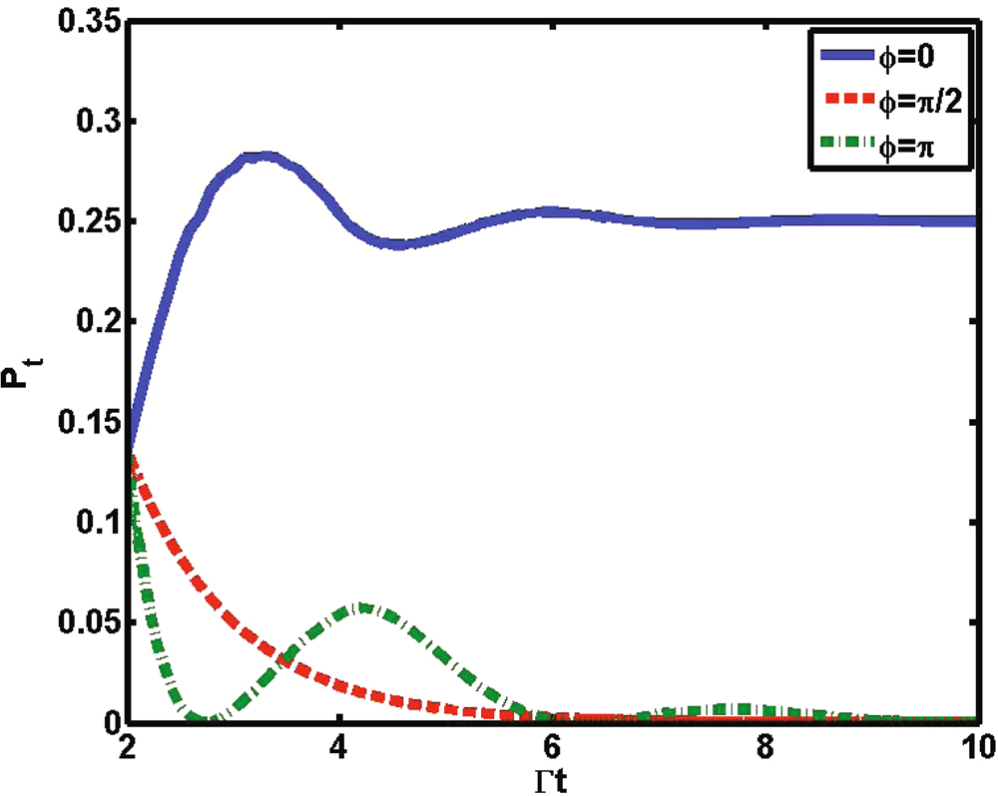
The atomic excited population *P*_*t*_ as a function of Γ*t* for various values of phase *ϕ* with Γ*t*_*d*_ = 2, *β* = 1, Δ = 0 and Ω = 0. Note that only the range *t* > *t*_*d*_ is shown due to the fact that the behavior exhibits an exponential decay and does not dependent on *ϕ* in *t* < *t*_*d*_.

The system-environment bound state is actually the stationary state of the entire system^42^. The formation of the bound state can lead to the inhibition of spontaneous emission, i.e., the system holds an amount of excitation in a long time. Furthermore, the stronger the bound state is, the greater the amount of excitation bounded around the system. This phenomenon has been demonstrated in super-Ohmic^43^ and PC baths^44^. For our two-level atomic system, the formation of the bound state can be detected by the excited-state population *P*_*t*_^45^, which is diagrammed in Fig. 4. Obviously, it can be confirmed that, if *ϕ* = *π*/2, population decreases monotonically to zero, implying that the bound state is absent. However, if *ϕ* = 0, part of the atomic excitation remains trapped. This trapping denotes the formation of the system-environment bound state. In the case *ϕ* = *π*, the bound state is established,but it is not stronger than the case in which *ϕ* = 0. Thus, *P*_*t*_ exhibits a periodic decrease; that is, only a small amount of excitation is reflected back to the qubit in this case.

In Fig. 2(b), we present the behaviors of non-Markovianity ℵ. The measure of non-Markovianity for our model is shown in the Appendix. We can find that the non-Markovianity connects directly with the formation of the bound state. Taking the situation in which Γ*t*_*d*_ = 2 [solid line in Fig. 2(b)] as an example, when the bound state is established for *ϕ* = *π*, the system presents a non-Markovian effect with ℵ = 0.06. At *ϕ* = 0, stronger bound states are created (the dynamics for *ϕ* = 0 is always Non-Markovian regardless of Γ*t*_*d*_ = 2^27^), and thus the non-Markovianity increases to ℵ = 0.16 from ℵ = 0.06. However, once the bound state is absent with *ϕ* = *π*/2, the behavior of sudden transition from non-Markovian to Markovian effect (ℵ = 0) occurs. Regarding the cases in which the atom is located close to the mirror (Γ*t*_*d*_ = 0.2) and far away from the mirror (${\rm{\Gamma }}{t}_{d}\gg 1$), the system-environment bound state will not be formed. Thus, the dynamics for Γ*t*_*d*_ = 0.2 and ${\rm{\Gamma }}{t}_{d}\gg 1$ can be considered Markovian. This result confirms the previous result that the non-Marikovianity is attributed to the formation of bound states^14^.

We now focus on the speed of quantum evolution. When the bound state is established and becomes stronger, the Markovian approximation of the environment fails and one might expect the memory effect to accelerate the speed of evolution. This would be true if one were considering a simple model in which the qubit is directly connected to a reservoir taking Lorentzian or Ohmic structures, as shown in ref.^18^. However, this relation may not be universally true. When we consider our much more complex physical model, in which a classical driven qubit is confined in a controllable photonic waveguide, a particularly astonishing phenomenon occurs. By comparing Fig. 2(a,b), one sees that the speed *V*_*a*_ and the non-Markovianity ℵ exhibit a non-monotonic relation. Here, we also take the case Γ*t*_*d*_ = 2 as an example. The particularly astonishing phenomenon is that when the non-Markovianity is the largest, ℵ = 0.16 (*ϕ* = 0), we find that the value of the speed is *V*_*a*_ = 0.078. In the cases ℵ = 0 (*ϕ* = *π*/2) and ℵ = 0.06 (*ϕ* = *π*), however, we can acquire *V*_*a*_ = 0.088 and 0.128, respectively. Importantly, one has to single out the special point *ϕ* = *π*/2 (or *ϕ* = 3*π*/2) in which the dynamical speeds for all regimes are equal. This may be because of the non-Markovianity for the three cases (Γ*t*_*d*_ = 0.2, 2, and $\gg 1$) with the same zero value (for *ϕ* = 0, the non-Markovvianity is not always zero^27^). However, not all of the points at which ℵ = 0 have the same dynamical speed *V*_*a*_, as shown in Fig. 2(b).

Thus, the surprising message is that a stronger system-environment bound state may not always be helpful in enhancing the speed of quantum evolution. What is the mechanism of speedup in a memory environment? What can be seen as an essential reflection to the speedup of quantum evolution? To answer these questions, in the next section we further investigate the speedup from the perspective of the direction of flow of information in memory environments.

### Mechanism for controlling speedup of quantum evolution

The non-Markovian effect of environment is tightly connected with the flow direction of information. This is because the accepted notion of non-Markovianity is based on the idea that the environment would cause the information backflow from environment to the system for non-Markovian process, while for a Markovian process the information flows in only one direction; that is, from the system to the environment, with no feedback^21^. The flow direction of information can be monitored by the changing rate of the trace distance, i.e., $\sigma (t,{\rho }_{\mathrm{1,2}}(0))=\frac{d}{dt}D({\rho }_{1}(t),{\rho }_{2}(t))$. The rate *σ*(*t*, *ρ*_1,2_(0)) is positive for an information backflow from the environment to the system, and negative for the information flowing in the opposite direction. Based on this, the total amount of backflow information $\aleph =\mathop{{\rm{\max }}}\limits_{{\rho }_{\mathrm{1,2}}(0)}{\int }_{\sigma \mathrm{ > 0}}dt\sigma (t,{\rho }_{\mathrm{1,2}}(0))$ is defined as the degree of non-Markovianity (see Method).

Previous studies have shown that a non-Markovian effect can speed up quantum evolution. However, the degree of non-Markovianity ℵ could not be seen as an essential reflection to the quantum speedup. That is to say, the reason for the speedup is not solely the backflow information. One question naturally arises: What is the effect of the information flowing from the system to the environment on quantum evolution?

**Figure 5:**
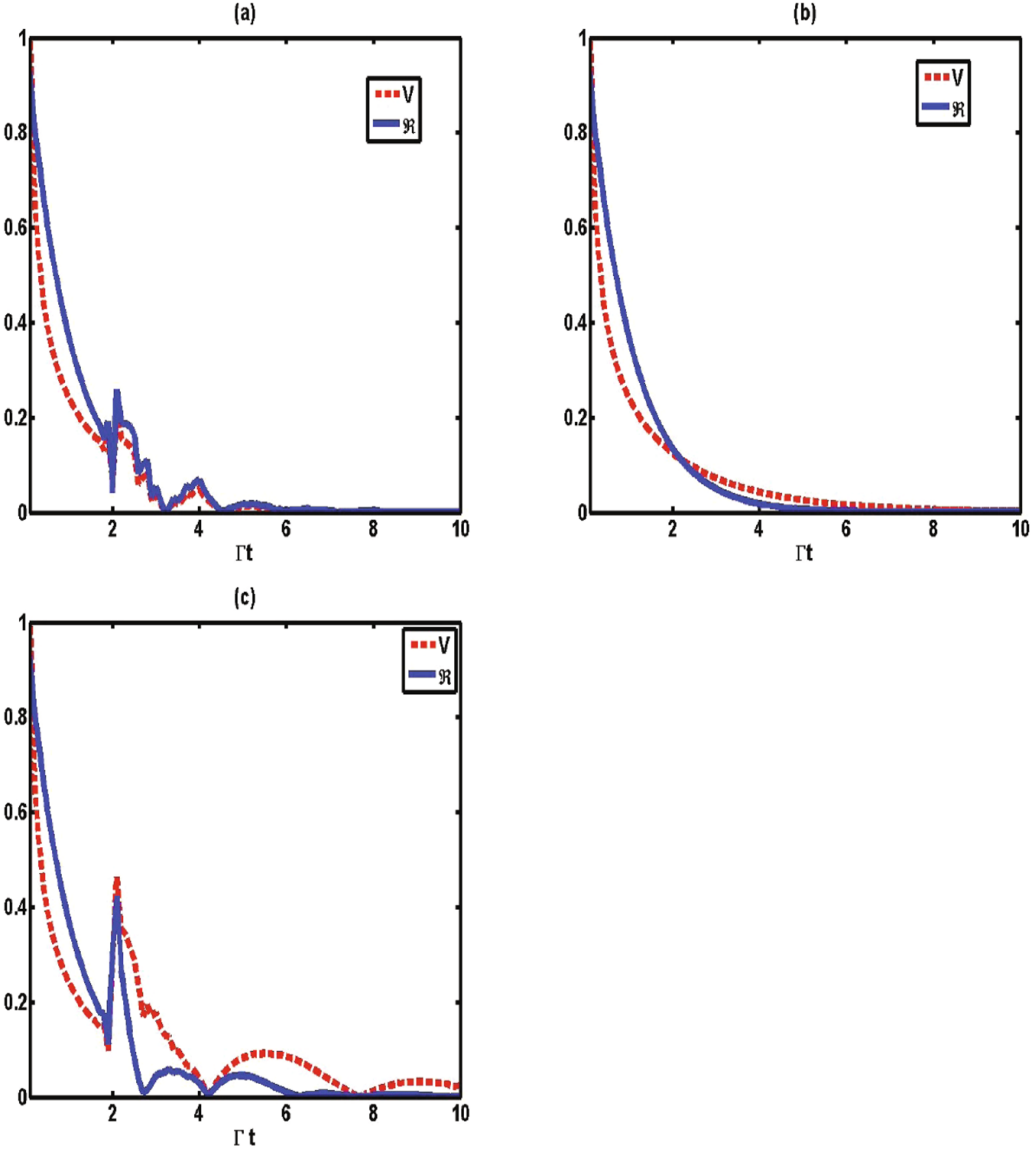
The instantaneous speed *V* (dashed line) and the absolute value of information changing rate $\Re =|\sigma (t,{\rho }_{\mathrm{1,2}}(0))|$ (solid line) as a function of Γ*t* for (**a**) *ϕ* = 0, (**b**) *ϕ* = *π*/2 and (**c**) *ϕ* = *π* with Γ*t*_*d*_ = 2, Ω = 0 and Δ = 0.

**Figure 6:**
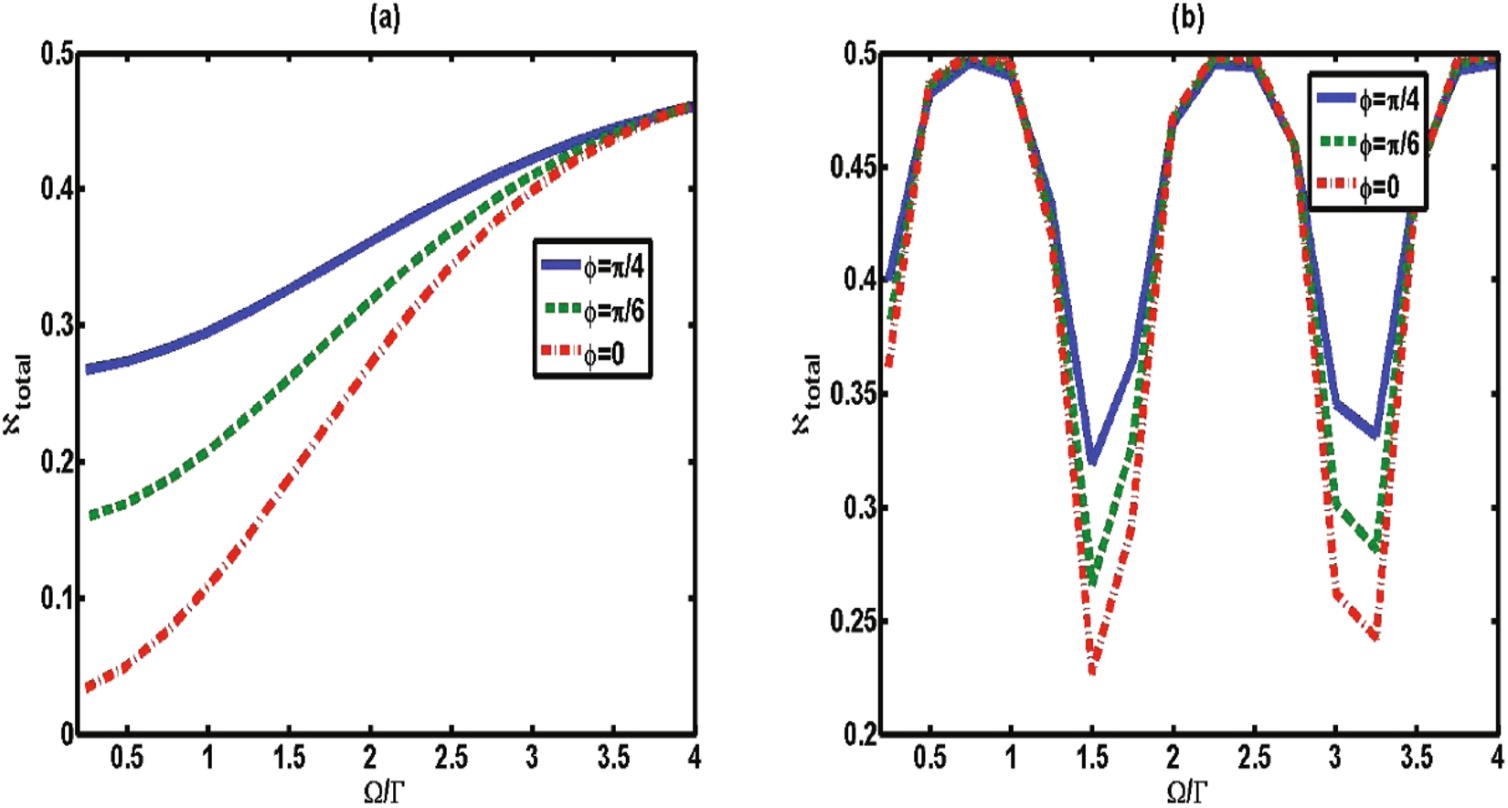
The total amount of flow information ℵ_*total*_ between the time zero and the time Γ*t* = 10 as a function of classical driving strength Ω for various values of phase *ϕ* with Δ = 0. (**a**) The memory time is small with Γ*t*_*d*_ = 0.2. (**b**) The memory time is intermediate with Γ*t*_*d*_ = 2.

Next, we focus on this question. In terms of above analysis, the total amount of flow information consisting of the flow from the system to the environment and the reverse flow is determined by:

22 $${\aleph }_{total}=\mathop{{\rm{\max }}}\limits_{{\rho }_{\mathrm{1,2}}(0)}\int dt\Re (t),$$

where the absolute value of the changing rate $\Re (t)=|\sigma (t,{\rho }_{\mathrm{1,2}}(0))|$ denotes the flow of information. The comparison of the changing rate $\Re (t)$ and the instantaneous speed *V* for various values of phase *ϕ* with a fixed memory time is shown in Fig. 5. It is interesting to find that the changing rate $\Re (t)$ exhibits the same behavior as the speed of quantum evolution; that is, the increase (decrease) of $\Re (t)$ leads to an increase (a decrease) of the instantaneous speed of quantum evolution. We thus conjecture that the flow of information plays a key role in controlling the speed of quantum evolution. To further study the mechanism of controllable speeding up of the evolution within the considered model, in Fig. 6 we plot the total amount of flow information, ℵ_*total*_, as a function of classical driving strength Ω for various values of phase *ϕ*. By contrasting the ℵ_*total*_ and the average speed shown in Fig. 3, the results also confirm that the driving strength can increase the information flow volume ℵ_*total*_, and thus accelerate the quantum speed of evolution. We therefore reach the interesting conclusion that it is the flow of information that directly affects the quantum speed of evolution, regardless of the direction of information flows. This is why, in some cases, the Markovian process (ℵ = 0) can also enhance the speed of evolution, as shown in Fig. 2(b).

## Discussion

We have studied a classically driven qubit that is coupled to a PC waveguide. We have investigated how the external classical driving strength and the reservoir’s memory time affect the quantum speed of evolution. We find that, with a judicious choice of the driving strength of the applied classical field, the speedup of evolution can be achieved in both Markovian (the memory time is small) and non-Markovian (the memory time is intermediate) processes. We have also explored the mechanism of well-controlled quantum speedup in our model. Surprisingly, a stronger system-reservoir bound state with a higher degree of non-Markovianity does not necessarily result in a greater speed of quantum evolution. More specifically, within the considered model, we have shown that it is not the amount of backflow information, i.e., the non-Markovianity, but rather the total amount of flow information that directly affects the average evolution speed for some interval of time. Our study sheds further light on the interplay between information flow and the evolution speed of an open quantum system.

Finally, it should be noted that our conclusion is not limited to the above model, but applies to the Jaynes-Cummings (JC) model for a two-level atom interacting with a lossy cavity featuring a Lorentzian spectral density^6^. Our model, in contrast, corresponds to a two-level atom coupled to a half-cavity, i.e., a cavity with one mirror^46^. For the JC model, the dynamical is characterized by two parameters: the spectral width *λ* and the atom-cavity coupling strength *γ*_0_. When the coupling is weak, i.e., *γ*_0_ < *λ*/2, the dynamics is Markovian, while for strong coupling with *γ*_0_ > *λ*/2 the dynamics converges to a non-Markovian setting. The population *P*_*t*_ exhibits a monotonic decay (Markovian dynamics) or a periodic decrease (non-Markovian dynamics). In addition, the population trapping will not occur^6,14,18^. The difference in speed between the Markovian and non-Markovian cases, i.e., the difference of the total amount of flow information (ℵ_*total*_) between them, is mainly determined by the backflow information. The effect of the information flowing from the system to the environment can be ignored. Thus, the non-Markovianity becomes the unique reason for quantum speedup in the JC model.

## Method

### Measure of non-Markovianity

In non-Markovian dynamics, the environment would cause the information backflow from the environment to the system. The non-Markovianity describing the total amount of backflow information is defined as:

23 $$\aleph =\mathop{{\rm{\max }}}\limits_{{\rho }_{\mathrm{1,2}}(0)}{\int }_{\sigma \mathrm{ > 0}}dt\sigma (t,{\rho }_{\mathrm{1,2}}(0)),$$

where $$\sigma (t,{\rho }_{\mathrm{1,2}}(0))=\frac{d}{dt}D({\rho }_{1}(t)\,and\,{\rho }_{2}(t))$$ denotes the changing rate of the trace distance $$D({\rho }_{1},{\rho }_{2}(t))=$$$$\frac{1}{2}tr|{\rho }_{1}(t)-{\rho }_{2}(t)|$$ between states *ρ*_1,2_(*t*) evolving from their respective initial states *ρ*_1,2_(0)^21^. The dynamical process is non-Markovian if a pair of initial states exists and exists at certain times such that *σ*(*t*, *ρ*_1,2_(0)) > 0. For our two-level system, the optimal pair of initial states has been proven to be $${\rho }_{\mathrm{1,2}}(0)=|\,\pm \,\rangle \langle \,\pm \,|$$^47^. Then, the trace distance can be acquired: *D*(*ρ*_1_(*t*), *ρ*_2_(*t*)) = |*c*_+_(*t*)|^2^.
